# Gastrointestinal ultrasonographic findings in cats with Feline panleukopenia: a case series

**DOI:** 10.1186/s12917-020-02720-w

**Published:** 2021-01-07

**Authors:** Isaya Rosaria, Ciccarelli Stefano, Enache Daniela, Specchi Swan, Pesaresi Marco, Ferri Filippo, Porporato Federico, Auriemma Edoardo, Contiero Barbara, Luigi M Coppola, Zini Eric

**Affiliations:** 1 Istituto Veterinario di Novara, 28060 Granozzo con Monticello (NO), Italy; 2 Department of Veterinary Medicine, University of Bari “Aldo Moro”, BA 70010 Valenzano, Italy; 3 Department of Animal Medicine, Production and Health, University of Padova, Legnaro, PD Italy; 4 Clinic for Small Animal Internal Medicine, Vetsuisse Faculty, University of Zurich, Winterthurerstrasse 260, 8057 Zurich, Switzerland

**Keywords:** Ultrasound, FPV, Cat, Gastrointestinal tract

## Abstract

**Background:**

Feline panleukopenia virus (FPV) is very resistant and highly contagious and infects domestic cats and other felids. FPV is particularly widespread among sheltered cats, and is associated with high morbidity and mortality, causing severe gastroenteritis characterized by anorexia, lethargy, fever, dehydration, hemorrhagic diarrhea, and vomiting. There is currently no data on the ultrasonographic features of cats affected with FPV. This case series describes abdominal ultrasonographic findings in shelter cats with naturally-occurring FPV, and assesses whether are associated with clinical and laboratory findings. Cats affected by FPV were enrolled in the study if an abdominal ultrasound was performed within 12 hours of diagnosis. Clinical, laboratory and survival data were collected from medical records. Ultrasonographic examinations were reviewed for gastrointestinal abnormalities and their associations with the above data were explored.

**Results:**

Twenty-one cats were included. Nine cats (42.9%) died and 12 (57.1%) recovered. Based on ultrasonography, the duodenum and jejunum showed thinning of the mucosal layer in 70.6% and 66.6% of cats, thickening of the muscular layer in 52.9% and 57.1% of cats, and hyperechogenicity of the mucosa in 41.2% and 33.3%. Jejunal hyperechoic mucosal band paralleling the submucosa and irregular luminal surface were both observed in 33.3% of the cats. Survival was positively associated with increased jejunal mucosal echogenicity (*P* = 0.003) and hyperechoic mucosal band (*P* = 0.003). Peritoneal free fluid was positively associated with vomiting (*P* = 0.002).

**Conclusions:**

This study provides ultrasonographic features of naturally-occurring FPV in cats, which, as expected, are compatible with gastroenteropathy. The most frequent findings were diffuse small intestine mucosal layer thinning, muscular layer thickening and mucosal hyperechogenicity, jejunal hyperechoic mucosal band and irregular luminal surface. Ultrasonographic features may be useful to complete the clinical picture and assess the severity of the gastroenteropathy in FPV cats. Prospective studies are needed to confirm ultrasonographic prognostic factors.

## Background

Feline panleukopenia virus (FPV) is a single-stranded, non-enveloped DNA virus that infects domestic cats and other felids as well as mink, raccoons, and foxes [1]. Replication of FPV occurs within mitotically active cells, such as intestinal crypt epithelial cells, damaging the intestinal villi, increasing permeability of the intestinal wall, and resulting in malabsorption [1, 2]. The most common clinical signs are anorexia, lethargy, fever, dehydration, hemorrhagic diarrhea, and vomiting [3]. Diagnosis is made by combining history, clinical findings, and hematologic changes together with detection of FPV or canine parvovirus (CPV) antigen in feces or viral DNA in blood or in feces [1, 4]. A recent study on survival and prognostic factors of FPV [5, 6] showed that shelter cats without signs of lethargy, with a higher body weight, or higher rectal temperature at admission were more likely to survive. Leukopenia on or after the third day of hospitalization was associated with poorer outcome. Treatment with amoxicillin-clavulanic acid, antiparasitics, or maropitant was associated with improved survival.

To the best of the authors’ knowledge, there is currently no information on the ultrasonographic features of the gastrointestinal tract of cats affected by FPV, especially regarding acute lesions. In a recent case report of an 8-month old kitten with FPV, a multi-layered appearance of the intestine was described representing superimposed wall layers and therefore resembling intussusceptions. However, a few hours after the ultrasonographic examination, the cat defecated a 15 cm-long piece of tissue and the histological examination revealed necrotic and cellular debris, indicating a fibrinonecrotic colonic cast [7–9].

The most frequent ultrasonographic abnormalities in dogs with CPV gastroenteritis include fluid and gas-filled gastrointestinal tract associated with generalized atony, decreased thickness of the mucosal layer, hyperechoic mucosal speckles, and irregularity of the luminal surface of the duodenum and jejunum [10, 11].

Gastrointestinal motility disorders are frequent in dogs and cats with acute vomiting or diarrhea. In cats with diffuse functional ileus associated with pancreatitis or abdominal effusion, corrugation of small bowel loops have been observed [9].

The aims of this retrospective study were therefore to: (i) describe abdominal ultrasonographic abnormalities in shelter cats with naturally-occurring panleukopenia; (ii) determine whether they are associated with clinical and laboratory findings; and (iii) investigate their impact on outcome.

## Results

Of the initial 265 cats with FPV infection, 21 (7.9%) met the inclusion criteria and were enrolled in the study. All cats were diagnosed with FPV based on clinical findings, laboratory abnormalities, and positive fecal antigen test (SNAP Parvo, IDEXX Laboratories, Milan, Italy). Four cats (19%) had been vaccinated between 11 and 20 months before disease onset. All cats were domestic shorthair. A total of 11 cats were intact females (52.4%) and 10 were intact males (47.6%) with a median age of 3 months (range: 1.5–24). Twelve cats (57.1%) survived and 9 (42.9%) died. The 12 cats that survived were discharged on average five days (median: 5, range: 4–7) after admission; according to the shelter veterinarian all the surviving cats fully recovered. The nine cats that did not survive died on average after two days (median: 2; range: 1–3). None of the cats were euthanized.

In three cats, information on clinical status was not available. For the remaining 18 cats (85.7%), major clinical signs on admission were lethargy in 14 (77.7%), diarrhea in 10 (55.5%), and vomiting in four (22.2%). Clinical signs had been observed on average two days (median: 2, range: 1–4) before admission.

Serological testing for FIV antibody and FeLV antigen were performed in each cat and all were negative. Fecal examination was performed in four cats (19%) that had not been treated with antiparasitics: three were negative and one was positive for *Capillaria*, *Uncinaria*, *Giardia*, and *Toxocara*. The remaining cats had been treated with antiparasitics during the week before admission and fecal examination was not performed.

All cats, except two in which the potassium was not measured, had a complete hematological and biochemical profile at admission. Table 1 summarizes the main abnormalities in the hematological and biochemical profiles of the 21 cats. These abnormalities consisted of a reduction in the hematocrit in 15 cats (71.4%) (26%, range: 14.9–35%) and of the erythrocyte count in 13 cats (61.9%) (6.9 × 10^6^ µL , range: 4.5-9 × 10^6^ µL), leukopenia in 12 (57.1%) (3.8 × 10^3^ µL, range: 0.1–15.6 × 10^3^ µL), thrombocytopenia in 11 (52.4%) (136 × 10^3^ µL, range: 0-774 × 10^3^ µL), hypoproteinemia in 12 (57.1%) (5.8 g/dL, range: 3.8–73 g/dL), and hypoalbuminemia in 10 (47.6%) (2.7 g/dL, range: 1.3–3.8 g/dL). Hypokalemia and hypoglycemia were present in two (9.5%) (4.8 mmol/L, range: 3-5.8 mmmol/L) and in one (4.8%) (130 mg/dL, range: 58–361 mg/dL) of the 21 cats, respectively.

**Table 1:** Hematological and biochemical abnormalities in 21 cats with feline panleukopenia

	Median	Range	Normal range
**Hematocrit (%)**	26	14.9–35	28.2–52.7
**Erythrocytes (× 10**^**6**^ µL**)**	6.9	4.5-9	7.1–11.5
**Leukocytes (× 10**^**3**^ µL**)**	3.8	0.1-15.63	3.9–19
**Platelets (× 10**^**3**^ µL**)**	136	0-774	155–641
**Total proteins (g/dL)**	5.8	3.8–73	5.9–8.7
**Albumin (g/dL)**	2.7	1.3–3.8	2.7–4.4
**Potassium (mmol/L)**	4.8	3-5.8	3.3–5.8
**Glucose (mg/dL)**	130	58–361	63–140

Table 2 reports the ultrasonographic measurements of the overall wall and layer thickness of the stomach, duodenum and jejunum.

**Table 2:** Wall and layer thickness of the stomach, duodenum and jejunum in cats with feline panleukopenia

Stomach (18 cats)				Duodenum (17 cats)			Jejunum (21 cats)		
	**Median (mm)**	**Range (mm)**	**Normal range (mm)**^a^	**Median (mm)**	**Range (mm)**	**Normal range (mm)**^b^	**Median (mm)**	**Range (mm)**	**Normal range (mm)**^b^
**Wall**	2.3	0.7–6.3	2-4.4	2.2	1.2–3.8	1.8–2.5	2.1	0.6–3.2	2-2.7
**Mucosa**	0.8	0.2–2.6	n.a.	1	0.4–1.9	1.1–1.6	0.7	0.2–1.7	1-1.6
**Submucosa**	0.5	0.2–4.7	n.a.	0.4	0.1–1.4	0.3–0.4	0.4	0.2–0.8	0.3–0.4
**Muscularis**	0.5	0.1–0.8	n.a.	0.4	0.2–0.8	0.3–0.4	0.6	0.1–1.5	0.3–0.5
**Serosa**	0.2	0.1–0.4	n.a.	0.1	0.1–0.3	0.2–0.4	0.2	0.1–0.3	0.2–0.4

^a^Goggin et al., 2000; ^b^Di Donato et al., 2014; *n.a.* not available

Information on the evaluation of the stomach was reported in 18 of 21 cats (85.7%); in 6 (33.3%) the stomach was moderately-to-severely distended by echogenic material and/or fluid, and in 12 (66.6%) cats it was normally distended. The median of the overall wall thickness was 2.3 mm (range: 0.7–6.3).

Information on the duodenum was available for 17 out of 21 cats (81%) and on the jejunum for all of the cats. The overall wall thickness was reduced in two cats (11.8%) in the duodenum (median: 2.2 mm, range: 1.2–3.8 mm) and in eight cats (38.1%) in the jejunum (median: 2.1 mm, range: 0.6–3.2 mm). Mucosal layer thickness of the duodenum (median: 1 mm, range: 0.4–1.9 mm) and of the jejunum (median: 0.7 mm, range: 0.2–1.7 mm) were reduced in 12 cats (70.6%) and in 14 cats (66.6%), respectively.

**Fig. 1:**
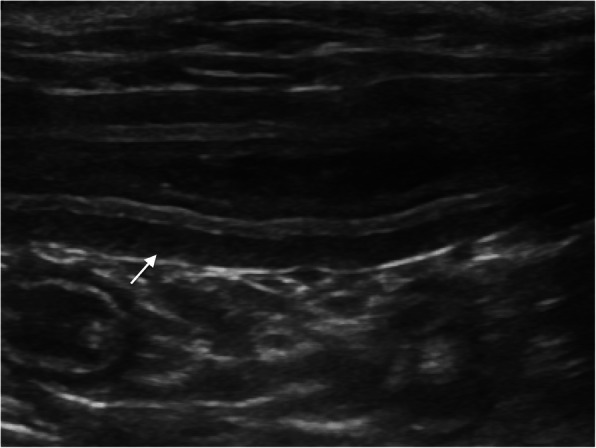
Thickening of the muscular layer in the jejunum

**Fig. 2:**
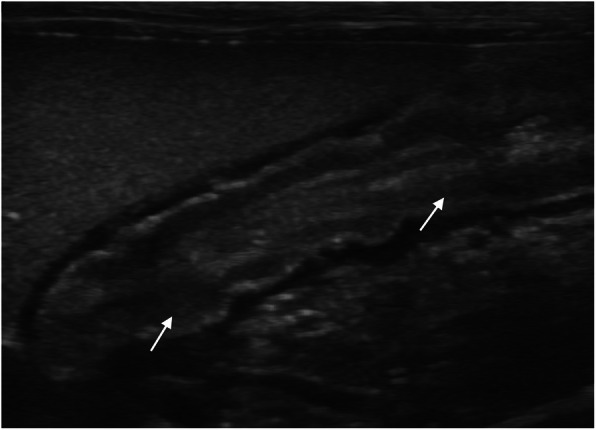
Increased echogenicity of the mucosa in the duodenum

**Fig. 3:**
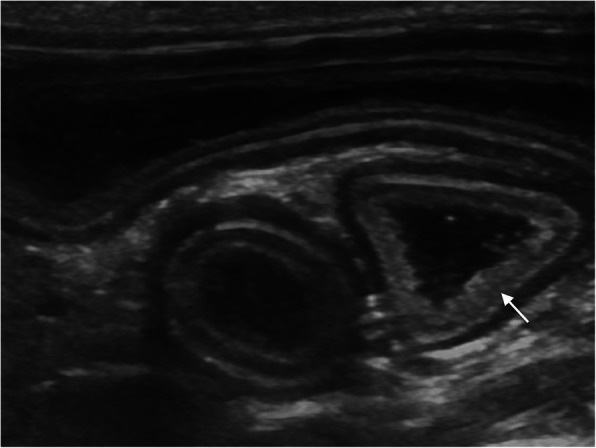
Increased echogenicity of the mucosa in the jejunum

**Fig. 4:**
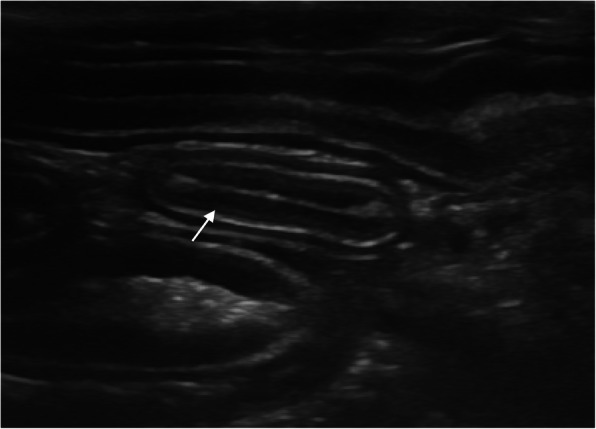
Hyperechoic linear mucosal band paralleling the submucosa in the jejunum

Thickening of the muscular layer was present in the duodenum (median: 0.4 mm, range: 0.2–0.8 mm) and jejunum (median: 0.6 mm, range: 0.1–1.5 mm) in nine cats (52.9%) and in 12 cats (57.1%), respectively (Insert Fig. 1). Increased echogenicity of the mucosa was detected in seven cats (41.2%) in the duodenum and in seven (33.3%) in the jejunum (Insert Figs. 2 and 3). Hyperechoic mucosal band paralleling the submucosa was present only in the jejunum of seven cats (33.3%) (Insert Fig. 4). The duodenum and jejunum were distended by fluid and/or echogenic material in two cats (11.8%) and in nine cats (42.8%), respectively. The luminal surface appeared irregular in two cats (11.8%) in the duodenum, and in seven (33.3%) in the jejunum; in these cases the finding was generalized.

The colon was evaluated in 15 of the 21 cats (71.4%), and the wall was considered thickened (thickness: >1.5 mm) in three cats (20%); one cat showed loss of layering.

Jejunal lymph nodes were described in 12 cats (57.1%), and the thickness was deemed normal (median: 4.6 mm, range: 3-6.2 mm). Images of the spleen were available in 18 cats (85.7%) and only in one cat was it considered enlarged (thickness: >9 mm); in seven cats (38.8%) the spleen had a “moth-eaten” parenchyma, and in three (16.6%) it was hypoechoic.

Five cats (23.8%) presented moderate anechoic abdominal effusion. Other findings recorded were hyperechogenicity of the mesentery in two cats with no abdominal effusion, multiple bilateral renal cysts in one, and hyperechogenicity of the liver parenchyma with hypoechoic multiple nodules, cystitis and peri-pancreatic steatitis in another.

No significant association was identified among the following parameters assessed with ultrasonography: duodenal wall thickness, duodenal muscularis thickness, jejunal wall thickness, jejunal muscularis thickness, duodenal mucosa hyperechogenicity, jejunal mucosa hyperechogenicity, jejunal hyperechoic mucosal band paralleling the submucosa, and peritoneal free fluid.

When associations were explored between ultrasonographic abnormalities and signalment, clinical findings, laboratory abnormalities, and survival, positive associations were observed between jejunal wall thickening and age (*P* < 0.001), as well as the presence of peritoneal effusion and vomiting (*P* = 0.002). A positive significant association was observed between survival and increased echogenicity in the jejunal mucosa *(P =* 0.003), and survival and presence of hyperechoic mucosal band in the jejunum (*P* = 0.003).

No other statistically significant associations were found.

## Discussion

Our study highlights that in cats with acute FPV the gastrointestinal tract shows several ultrasonographic changes. The most frequent findings were diffuse small intestine mucosal layer thinning, muscular layer thickening and mucosal hyperechogenicity. Hyperechoic mucosal band paralleling the submucosa and irregular luminal surface were frequent in the jejunum.

Although described mostly in chronic enteropathies [12–16], our study identified diffuse small intestine muscular layer thickening, and hyperechoic mucosal band paralleling the submucosa, along with increased mucosal echogenicity in cats with acute gastroenteritis induced by FPV.

Thickening of the muscular layer was observed in the duodenum and jejunum in approximately 50% and 60% of cats, respectively, which has previously been associated with chronic enteropathies (e.g., eosinophilic enteritis), intestinal foreign body and lymphoma; potential causes are infiltrative diseases and smooth muscle hypertrophy or hyperplasia [12, 13, 15, 16]. No histopathological analysis was performed in our cats, and occult chronic enteropathy (e.g., food intolerance) or lymphoma cannot be rule out in our cases.

However, it seems unlikely that 60% of these young cats had an underlying disease. We assume that muscular layer thickening in cats with acute panleukopenia may be due to the inflammatory condition. Enteritis has been shown to cause diffuse intestinal wall thickening associated with edema, hemorrhage, fibrosis and/or necrosis in dogs [17]; these changes are expected to cause intestinal wall thickening also in cats, including the muscular layer.

In our study, the hyperechoic mucosal band paralleling the submucosa was present in the jejunum in one-third of the cats, while not in the duodenum. Using ultrasound, one study [14] described the presence of a mucosal hyperechoic band paralleling the submucosa in all cats with full-thickness intestinal biopsy presenting with gastrointestinal signs. Based on histology, the hyperechoic mucosal band likely represented an ultrasonographic interface due to the presence of mucosal fibrosis. In the same investigation [14], the authors prospectively evaluated clinical signs of cats showing a visible hyperechoic mucosal band on ultrasound but without performing histopathology. Most cats had vomiting or diarrhea, but one-third had no clinical signs or other ultrasonographic intestinal abnormalities. It was concluded that this band can also be observed in apparently healthy cats [14].

We observed increased mucosal echogenicity in approximately 40% of cats in the duodenum, and 30% in the jejunum. In addition, irregularity of the luminal surface was found in 10% of cats in the duodenum, and in 30% of cats in the jejunum. Mucosal changes, such as increased mucosal echogenicity, hyperechoic speckles, and striations have been reported in several conditions in chronic enteropathies in dogs and cats. Increased mucosal echogenicity has been described in lymphoplasmacytic enteritis and eosinophilic enteritis and in cats with mucosal fibrosis [14, 15]. Hyperechoic speckles and hyperechoic striations have been reported in dogs with chronic enteropathies [18]. A thick hyperechoic mucosal border on the luminal margin of several jejunal segments was observed in dogs with acute enteritis [19].

In our cats with FPV, it is possible that these findings were due to mucus, cellular debris, gas entrapped in the mucosal crypts, and protein accumulation caused by necrosis and inflammation associated with the acute form of the disease, as reported in puppies with CPV [10].

In our study, mucosal thinning of the duodenum and jejunum was observed in two-thirds of cases, along with an irregular luminal surface in two cats (11.8%) in the duodenum, and in seven (33.3%) cats in the jejunum. Villous atrophy secondary to viral-induced crypt cell destruction, necrosis, and sloughing of epithelial cells associated with FPV are reasonable explanations for the reduced thickness. This finding is similar to previous observations in 40 puppies with CPV where the duodenal and jejunal mucosa thickness was significantly decreased and the luminal surface of the duodenal and jejunal mucosa was irregular [10]. The thinning of the mucosal layer due to the aforementioned causes, could explain the severe reduction in the jejunal wall that we observed in one cat in which the thickness was 0.6 mm.

In cats with ultrasonographic information on the jejunal lymph nodes, echogenicity, echotexture, and size were normal [20]. This finding is similar to previous observations in puppies with CPV [10], in which parvovirus infection was not associated with jejunal lymphadenopathy by abdominal ultrasound. In the above mentioned study the authors assumed that cortical atrophy was the result of the tendency of CPV to rapidly divide in cells of the lymphoid tissue [10]. It is also possible that the same explanation accounts for the sonographically normal lymph nodes in our study, although histology would have been required for confirmation.

In almost half of the cats with available images of the spleen, a “moth-eaten” parenchyma was observed, and in only one was this finding associated with splenomegaly. A “moth-eaten” parenchyma is characterized by numerous small hypoechoic nodules spread throughout the organ causing a spotted echotexture. However, this finding is non-specific, and in a previous study was observed in cats with lymphoid hyperplasia, extramedullary hematopoiesis, passive congestion, lymphoma, carcinoma, and histiocytosis, as well as in healthy animals [21]. The use of very high-frequency transducers (11–18 MHz), which improve the quality of images, may also be partly responsible for the high rate of this particular pattern in our cats.

Regarding the associations between ultrasonographic features and clinical findings, vomiting was positively associated with free peritoneal fluid. Due to lack of gastrointestinal biopsies and fluid analysis, the cause for this association remains unclear. Mild abdominal effusion has been described in puppies with CPV, although associations with clinical signs were not investigated [10]. Age was also positively associated with jejunal wall thickening. However, the reason for this finding is not clear, hence a casual association rather than a cause-and-effect relationship cannot be excluded.

Interestingly, based on ultrasonography, the abdominal effusion of the present cats was anechoic. Feline infectious peritonitis seemed unlikely because in this disease the effusion is usually echogenic and with mobile particles [22]; it is still nevertheless possible. The fluid was not collected to clarify its pathogenesis.

With regard to survival and abdominal ultrasonography, a favorable outcome was positively associated with higher jejunal mucosal echogenicity and hyperechoic mucosal band in FPV cats. The hyperechoic mucosal band may represent an ultrasonographic interface due to the presence of mucosal fibrosis [14]. Unfortunately, histopathology was not performed in our cats with FPV to verify whether fibrous tissue was present in the jejunum. Given that FPV has an acute onset in most cats [3], the presence of fibrous tissue would be unexpected. However, in some cats FPV may be more subclinical, longer lasting and with a better outcome [1]. It is also possible that intestinal fibrosis develops in some of these cases.

Although to the author’s knowledge there is no study describing acute enteritis in FPV cats, thinning and hyperechogenicity of the mucosa, thickening of the muscular layer, reduction of the gastrointestinal motility and gastric and intestinal distention with fluid and/or echogenic material might yield information about the severity of the enteropathy in these patients; prospective studies are needed to test this hypothesis.

The main limitations of the present study are the relatively small number of cats included and the retrospective nature of the investigation. Some medical records were incomplete, in particular those pertaining to images and videos of the ultrasound. Hence, it was not possible to measure all segments of the gastrointestinal tract, although the attending radiologist reported them as normal.

Moreover, follow-up abdominal ultrasound and histological examinations were not performed, which would have characterized the underlying causes of some of the ultrasonographic features. Finally, no control group of shelter cats with other gastrointestinal diseases was available for comparison. However, shelter cats with vomiting or diarrhea but otherwise in good conditions are treated symptomatically by the shelter veterinarian without performing further diagnostics, such as abdominal ultrasound, thus precluding the possibility of a control group.

Collectively, the present ultrasonographic findings in FPV cats may be useful to characterize the enteritis and assess its severity, and to set the basis for further prospective studies.

## Conclusions

In brief, cats with naturally-occurring FPV had ultrasonographic features compatible with gastroenteropathy. Diffuse small intestine mucosal layer thinning, muscular layer thickening and mucosal hyperechogenicity, and jejunal hyperechoic mucosal band paralleling the submucosa and irregular luminal surface were frequently observed in the cats examined. Some ultrasonographic abnormalities were associated with clinical findings and outcome, although the underlying reasons have not been clarified. Prospective studies with a larger number of cases are expected to ameliorate the characterization of FPV enteritis and possibly confirm ultrasonographic prognostic factors observed in this study or by others [5], in order to improve treatment and outcome.

## Methods

### Cats and inclusion criteria

An outbreak of FPV infection occurred in a large cat shelter located in northwestern Italy at the end of autumn 2010 which persisted for more than three consecutive years, thus becoming endemic. Retrospectively, medical records of cats with FPV infection admitted to the authors’ institution between January 2011 and December 2013 were reviewed and information pertaining to signalment, history, clinical signs, and laboratory findings at presentation, as well as survival at discharge from the hospital were collected. Vaccination status, fecal examination, and whether cats had positive or negative results of anti-FIV antibody and FeLV antigen testing (SNAP FIV/FeLV combo plus test, IDEXX Laboratories, Milan, Italy) were also recorded [23].

In our study, cats were included if diagnosed with FPV based on compatible clinical findings (i.e., lethargy, diarrhea or vomiting), laboratory abnormalities (i.e., anemia, leukopenia, thrombocytopenia, hypoproteinemia, hypoalbuminemia, hypokalemia or hypoglycemia) and positive fecal antigen test (SNAP Parvo, IDEXX Laboratories, Milan, Italy), and if an abdominal ultrasound had been performed within 12 hours of admission. Fasting longer than eight hours was not possible because the sample included kittens and debilitated cats.

### Abdominal ultrasonography

Abdominal B-mode examination was performed in conscious cats placed in dorsal recumbency using a GE-LogiQ S8 (GE Healthcare, Milan, Italy) with a high-frequency transducer (11–18 MHz). Prior to ultrasound, hair was clipped on the abdominal area, and coupling gel was used. A board-certified radiologist (S.S.) along with a diagnostic imaging intern (R.I.) reviewed all ultrasound documentation. Cats were included if the quality of images was judged as adequate.

Ultrasound videos, static images and reports were reviewed to assess abdominal organs, with emphasis on the gastrointestinal tract. The ultrasonographic features collected from the gastrointestinal tract were: overall wall and layer thickness (e.g., muscular layer thickening), both measured in a transverse and sagittal axis from the inner hyperechoic interface of the mucosal surface to the outer hyperechoic layer of the serosa; echogenicity of the various layers (e.g., hyperechogenicity of the mucosa); luminal patterns (e.g., fluid-filled); morphologic description, location and extension of any intestinal lesion.

Layering of the intestinal wall was considered abnormal if layers had changes in echogenicity or in thickness, as described by previous authors (Table 2) [24, 25]. Peristalsis was reduced if peristaltic acts were ≤ 3/min and increased if > 5/min [26].

In addition, size, echogenicity, echotexture and morphology of jejunal lymph nodes and spleen, and the presence of peritoneal free fluid were evaluated. Any other abdominal abnormalities were recorded if present.

### Statistical analysis

Statistical analysis was performed using SAS v. 9.3 (SAS Institute Inc, Cary, NC). The median and range were calculated for the overall wall thickness and for individual layers of the gastrointestinal segments (e.g., stomach, duodenum, jejunum).

Several parameters were considered for this exploratory analysis. In particular, (i) signalment and clinical variables included age, presence of vomiting, diarrhea, lethargy, and survival; (ii) laboratory findings included erythrocytes, leukocytes, platelet count, serum concentration of total proteins, albumin, glucose, and potassium; and (iii) ultrasonography included duodenal wall thickness, duodenal muscularis thickness, jejunal wall thickness, jejunal muscularis thickness, duodenal mucosa hyperechogenicity, jejunal mucosa hyperechogenicity, jejunal hyperechoic mucosal band paralleling the submucosa, and peritoneal free fluid.

These clinical and laboratory variables were used to determine whether they were associated with ultrasonographic findings. Associations between ultrasonographic abnormalities and ultrasonographic, signalment, and clinical and laboratory data were explored using Chi-square and Fisher’s exact tests. Due to the numerous analyses, significance was corrected with Bonferroni, and set at *P* < 0.001 for associations between ultrasonographic and ultrasonographic or laboratory data, and set at *P* < 0.01 for associations between ultrasonographic and signalment or clinical data including survival [27].

To explore the above associations, all findings were categorized into: present vs. absent or abnormal vs. normal. Laboratory parameters, including erythrocytes, leukocytes, platelet count, total proteins, albumin, glucose, and potassium, were considered abnormal if lower than the reference interval. In addition, age was divided into < 1 year vs. >1 year. Cats were considered alive if they survived up to discharge from the hospital.

## Data Availability

The datasets used and/or analysed during the current study are available from the corresponding author upon reasonable request.

## Abbreviations

CPV: Canine Parvovirus
DNA: Deoxyribonucleic Acid
FeLV: Feline Leukemia Virus
FIV: Feline Immunodeficiency Virus
FPV: Feline Panleukopenia Virus
MHz: Megahertz

## References

[1] Stuetzer B, Hartmann K (2014). Feline parvovirus infection and associated diseases. Vet J.

[2] Truyen U, Addie D, Belák S (2009). Feline panleukopenia: ABCD guidelines on prevention and management. J Feline Med Surg.

[3] Greene CE. Feline enteric viral infections. Infectious diseases of the dog and cat. 2012:80–91.

[4] Litster A, Benjanirut C (2014). Case series of feline panleukopenia virus in an animal shelter. J Feline Med Surg.

[5] Porporato F, Horzinek MC, Hofmann-Lehmann R (2018). Survival estimates and outcome predictors for shelter cats with feline panleukopenia virus infection. J Am Vet Med Asso.

[6] Kruse BD, Unterer S, Horlacher K, Sauter-Louis C (2010). Prognostic factors in cats with feline panleukopenia. J Vet Intern Med.

[7] Lee H, Lee M, Lee J (2012). Ultrasonographic diagnosis of a fibrinonecrotic colonic cast in a kitten with feline panleukopenia virus. J Small Anim Pract.

[8] Penninck D, Nyland TG, Kerr LY, Fisher PE (1990). Ultrasonographic evaluation of gastrointestinal diseases in small animals Vet Radiol Ultrasound.

[9] Moon ML, Biller DS, Armbrust LJ (2003). Ultrasonographic appearance and etiology of corrugated small intestine. Vet Radiol Ultrasound.

[10] Stander N, Wagner WM, Goddard A (2010). Ultrasonographic appearance of canine parvoviral enteritis in puppies. Vet Radiol Ultrasound.

[11] Mylonakis ME, Kalli I, Rallis TS (2016). Canine parvoviral enteritis: an update on clinical diagnosis, treatment, and prevention. Vet Med: Res Reports.

[12] Diana A, Pietra M, Guglielmini C (2003). Ultrasonographic and pathologic features of intestinal smooth muscle hypertrophy in four cats. Vet Radiol Ultrasound.

[13] Daniaux LA, Laurenson MP, Marks SL (2014). Ultrasonographic thickening of the muscularis propria in feline small intestinal small cell T-cell lymphoma and inflammatory bowel disease. J Feline Med Surg.

[14] Penninck DG, Webster CR, Keating JH (2010). The sonographic appearance of intestinal mucosal fibrosis in cats. Vet Radiol Ultrasound.

[15] Tucker S, Penninck DG, Keating JH, Webster CRL (2014). The clinicopathological and ultrasonographic features of cats with eosinophilic enteritis. J Feline Med Surg.

[16] Zwingenberger AL, Marks SL, Baker TW, Moore PF (2010). Ultrasonographic evaluation of the muscularis propria in cats with diffuse small intestinal lymphoma or inflammatory bowel disesase. J Vet Intern Med.

[17] Penninck D, Smyers B, Webster CRL (2003). Diagnostic value of ultrasonography in differentiating enteritis from intestinal neoplasia in dogs. Vet Radiol Ultrasound.

[18] Gaschen L (2011). Ultrasonography of small intestinal inflammatory and neoplastic diseases in dogs and cats. Vet Clin North Am Small Anim Pract.

[19] Penninck D, d’Anjou MA. Gastrointestinal Tract. Atlas of Small Animal Ultrasonography. 2015:259–08.

[20] Schreurs E, Vermote K, Barberet V (2008). Ultrasonographic anatomy of abdominal lymph nodes in the normal cat. Vet Radiol Ultrasound..

[21] Bertal M, Carmel EN, Diana A (2018). Associations between ultrasonographic appearance of splenic parenchyma and cytology in cats. J Feline Med Surg.

[22] Lewis KM, O’Brien RT (2010). Abdominal ultrasonographic findings associated with feline infectious peritonitis: a retrospective review of 16 cases. J Am Anim Hosp Assoc.

[23] Abd-Eldaim M, Beall MJ, Kennedy MA. Detection of feline panleukopenia virus using a commercial ELISA for canine parvovirus. Vet Ther. 2009 Winter;10(4):E1–6. 20425728

[24] Di Donato P, Penninck DG, Pietra M (2014). Ultrasonographic measurement of the relative thickness of the intestinal wall layers in clinically healthy cats. J Feline Med Surg.

[25] Goggin JM, Biller DS, Debey BM, et al. Ultrasonographic measurement of gastrointestinal wall thickness and the ultrasonographic appearance of the ileocolic region in healthy cats. J Am Anim Hosp Assoc. 2000;36:224–8. 10.5326/15473317-36-3-224 10825093

[26] Dressman JB (1986). Comparison of canine and human gastrointestinal physiology. Pharm Res.

[27] Bender R, Lange S (2001). Adjusting for multiple testing – when and how?. J Clin Epidemiol.

