# Correction to: Gastrointestinal ultrasonographic findings in cats with Feline panleukopenia: a case series

**DOI:** 10.1186/s12917-021-02856-3

**Published:** 2021-04-02

**Authors:** Rosaria Isaya, Stefano Ciccarelli, Daniela Enache, Swan Specchi, Marco Pesaresi, Filippo Ferri, Federico Porporato, Edoardo Auriemma, Barbara Contiero, Luigi M. Coppola, Eric Zini

**Affiliations:** 1 Istituto Veterinario di Novara, 28060 Granozzo con Monticello (NO), Italy; 2 Department of Veterinary Medicine, University of Bari “Aldo Moro”, 70010 Valenzano, BA Italy; 3 Department of Animal Medicine, Production and Health, University of Padova, Legnaro, PD Italy; 4 Clinic for Small Animal Internal Medicine, Vetsuisse Faculty, University of Zurich, Winterthurerstrasse 260, 8057 Zurich, Switzerland

**Correction to: BMC Vet Res 17, 20 (2021)**

**https://doi.org/10.1186/s12917-020-02720-w**

The original article [1] incorrectly inverted several author names. This error has since been corrected.
